# Nitrogen removal processes in lakes of different trophic states from on-site measurements and historic data

**DOI:** 10.1007/s00027-021-00795-7

**Published:** 2021-03-10

**Authors:** Beat Müller, Raoul Thoma, Kathrin B. L. Baumann, Cameron M. Callbeck, Carsten J. Schubert

**Affiliations:** Eawag, Swiss Federal Institute of Aquatic Science and Technology, 6047 Kastanienbaum, Switzerland

**Keywords:** Nitrogen removal rate, Nitrogen removal efficiency, Combined nitrification–denitrification, Sediment porewater fluxes, Whole-lake nitrogen budget

## Abstract

Freshwater lakes are essential hotspots for the removal of excessive anthropogenic nitrogen (N) loads transported from the land to coastal oceans. The biogeochemical processes responsible for N removal, the corresponding transformation rates and overall removal efficiencies differ between lakes, however, it is unclear what the main controlling factors are. Here, we investigated the factors that moderate the rates of N removal under contrasting trophic states in two lakes located in central Switzerland. In the eutrophic Lake Baldegg and the oligotrophic Lake Sarnen, we specifically examined seasonal sediment porewater chemistry, organic matter sedimentation rates, as well as 33-year of historic water column data. We find that the eutrophic Lake Baldegg, which contributed to the removal of 20 ± 6.6 gN m^−2^ year^−1^, effectively removed two-thirds of the total areal N load. In stark contrast, the more oligotrophic Lake Sarnen contributed to 3.2 ± 4.2 gN m^−2^ year^−1^, and had removed only one-third of the areal N load. The historic dataset of the eutrophic lake revealed a close linkage between annual loads of dissolved N (DN) and removal rates (NRR = 0.63 × DN load) and a significant correlation of the concentration of bottom water nitrate and removal rates. We further show that the seasonal increase in N removal rates of the eutrophic lake correlated significantly with seasonal oxygen fluxes measured across the water–sediment interface (R^2^ = 0.75). We suggest that increasing oxygen enhances sediment mineralization and stimulates nitrification, indirectly enhancing denitrification activity.

**Supplementary Information:**

The online version contains supplementary material available at 10.1007/s00027-021-00795-7.

## Introduction

Increasing anthropogenic nitrogen (N) loading in agriculturally dominated terrestrial systems has the potential to induce significant eutrophication in downstream aquatic environments and coastal marine ecosystems where N is often a key nutrient limiting primary productivity (Howarth and Marino 2006; Diaz and Rosenberg 2008; Howarth et al. 2011; Erisman et al. 2013). In the transport pathway from land to the ocean, lakes play an essential role with respect to the removal of N. Current estimates indicate that lakes contribute to the removal of about one-third of total N that enters surface freshwaters globally (Harrison et al. 2009). Small lakes (< 50 km^2^) with oxic overlying waters are especially important as these systems sustain active N turnover at the oxic–anoxic interface in the upper sediments (Seitzinger et al. 2006).

At the oxic–anoxic interface, the removal of fixed N is governed by a complex interplay of microbial transformation processes that operate in consortium to transform organic- and inorganic-N to produce N_2_ gas, thereby removing bioavailable N from the ecosystem. In aquatic environments, including marine and freshwater sediments the N cycle comprises of N_2_ fixation, denitrification, nitrification, anammox (Kuypers et al. 2018; Crowe et al. 2017), dissimilatory nitrate (NO_3_^−^) reduction to ammonium (DNRA, Giblin et al. 2013; Burgin and Hamilton 2007), comammox (Daims et al. 2015; van Kessel et al. 2015), and organic nitrogen remineralization to ammonium.

Studies in permeable marine sediments, where nitrification–denitrification are tightly coupled, posit that the availability of oxygen (O_2_) may play a role in moderating denitrification. For instance, the presence of O_2_ supports the mineralization of organic matter to ammonium (Marchant et al. 2016) that may drive enhanced aerobic/microaerobic nitrification activity in coastal sediments (Laursen and Seitzinger 2002; Marchant et al. 2016). However, as lake sediments are generally less permeable, nitrification–denitrification coupling may be less pronounced or possibly negligible in some cases, thus it remains unclear if O_2_ availability could have an effect on N loss. Indeed, very few studies have investigated this relationship in less O_2_ permeable freshwater lake sediments (e.g. Li and Katsev 2014; Rissanen et al. 2011). In contrast, N removal rates in lakes receiving high inputs of dissolved N decreased in the presence of O_2_, and the concentration of NO_3_^−^ in the sediment overlying water was the main source for denitrification while coupled nitrification–denitrification was negligible (Höhener and Gächter 1993; Pina-Ochoa and Alvarez-Cobelas 2006; Alvarez-Cobelas et al. 2019).

Hitherto studies in lakes observed that the N removal rate (NRR) increased with increasing areal load (David et al. 2006; Finlay et al. 2013) and that the removal efficiency (the fraction removed from the total N load, NRE) depended on the water retention time of the lake (Kelly et al. 1987; Howarth et al. 1996; Saunders and Kalff 2001a; Seitzinger et al. 2006). Finlay et al. (2013) reported observations indicating that decreasing concentrations of bio-available P in lakes led to a subsequent decrease of N removal rates. This might occur due to decreased assimilation and settling, declining reducing conditions in lake sediments and therefore diminishment of lake areas efficient in the removal of N.

In this study, we explored and quantified the seasonality of N removal rates and the effect of the presence of O_2_ and NO_3_^−^ at the sediment–water interface of two freshwater lakes of contrasting trophic states. Using 33 years of historic data allowed estimating N removal efficiency and to relate it to N loads from the catchment. Finally, we discuss the effect of O_2_ on N removal, providing a better understanding of the factors that govern N loss under two contrasting trophic scenarios. The findings expand our understanding of the driving forces controlling removal rates and removal efficiency promoting N transformation in lakes and enlighten the ambiguous role of O_2_.

## Methods

### Study sites

**Fig. 1:**
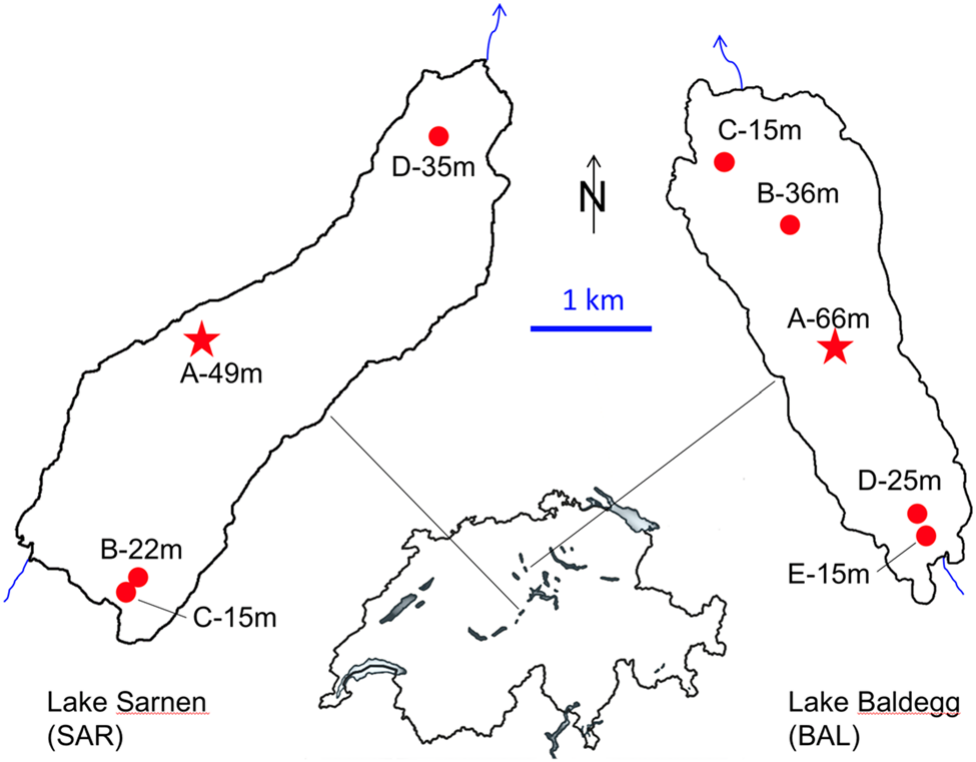
Maps of eutrophic Lake Baldegg (BAL) located on the Swiss Plateau, and oligotrophic Lake Sarnen (SAR) in the peri-alpine region of Switzerland. Porewater nutrients and O_2_ concentrations were monitored from sediment cores collected at all sampling stations indicated in red. Positions of the moorings with sediment traps and locations of additional monitoring of water column chemistry collected at the deepest station are indicated by the red star (color figure online)

Lakes Sarnen and Baldegg, 7.15 and 5.22 km^2^ in size, respectively, are classified as small lakes according to Seitzinger et al. (2006). Both lakes are monomictic with complete turnover between mid-November to end of March, and always ice-free. They are thermally stratified between April and October to mid-November. Lake Baldegg, with maximum water depth of 66 m, is located on the Swiss Plateau and is heavily impacted by P and N loads as a consequence of the agriculturally dominated catchment region (i.e. cattle and pig breeding farms; Fig. 1). Phytoplankton is dominated by the cyanobacterium *Planktothrix rubescens*. Over the past few decades, the lake has undergone a remediation phase, which has curtailed water column total P concentrations from 520 mg P m^−3^ in the 1970s to ~ 24 mg P m^−3^ today (Müller et al. 2019). Moreover, peak N was observed in the 1980s with up to 2.3 g N m^−3^ (Wehrli et al. 1997). Despite efforts to mitigate the P and N backgrounds, Lake Baldegg is still eutrophic and sustains high primary productivity in the summer. To prevent complete bottom water anoxia from developing, the hypolimnion has been artificially aerated during the stratified season with up to 3 t O_2_ day^−1^ since 1984 (Gächter and Wehrli 1998), thus, the waters overlying the sediments contain O_2_. In contrast, Lake Sarnen, with a maximum water depth of 49 m is located in the pre-Alps in a more mountainous catchment region that is more densely forested and only extensively farmed. The catchment area is over threefold larger than Lake Baldegg (Table 1), and thereby Lake Sarnen receives high loads of allochthonous material, including occasional storm-water events. Unlike Lake Baldegg, Lake Sarnen has remained oligotrophic with TP- and dissolved inorganic N (DIN) concentrations of ~ 5 mg P m^−3^ and ~ 0.56 g N m^−3^, respectively, and the plankton community dominated by diatoms.

**Table 1:** Orographic data of Lakes Baldegg and Sarnen and sampling protocol

Lake	Baldegg	Sarnen
Volume [m^3^]	1.74 10^8^	2.41 10^8^
Surface area [m^2^]	5.22 10^6^	7.15 10^6^
Hypolimnion area (15 m depth) [m^2^]	4.10 10^6^	5.79 10^6^
Catchment area [km^2^]	73.3	234
Water discharge [m^3^ s^−1^]	1.24	9.61
Residence time [year]	4.3	0.76
Max. depth [m]	66	49
Trophic state	Eutrophic (24 mg TP m^−3^)	Oligotrophic (~ 5 mg TP m^−3^)
Cores and sampling locations	BAL A: 66 m, deepest point BAL B: 36 m, betw. center and outlet BAL C: 15 m, near outlet BAL D: 25 m, near inlet, delta region BAL E: 15 m, near inlet, delta region	SAR A: 49 m, deepest point SAR B: 22 m, Inlet, delta region SAR C: 15 m, Inlet, delta region SAR D: 35 m, intermediate, near outlet
Sampling dates	Jun 17, Oct 17, Mar 18, May 18, Jun 18, Aug 18, Sept 18. (BAL D not meas in Jun 17)	Jun 17, Oct 17, Mar 18, May 18, Jun 18, Aug 18, Sept 18. (SAR D not meas in Jun 17)

### Sediment sampling, porewater nutrient and oxygen analysis

We used a gravity corer equipped with a PVC tube (60 cm length and 5.9 cm inner diameter, uwitec, Austria) for sediment sampling. The PVC tube was pre-drilled with four parallel columns of holes (1.2 mm diameter with 10 mm vertical resolution) resulting in a vertical resolution of 2.5 mm. The PVC holes were sealed with adhesive tape prior to sampling. Sediment cores were recovered from Lakes Baldegg and Sarnen between June 2017 and September 2018, with a specific focus on the spring–summer period (Table 1). Over this period, one core was recovered from 4 to 5 sampling stations each, situated near the lake inflow, near the center of the lake, and near the lake outflow (Table 1). Intact cores were transported to an on-site facility for immediate processing. Sediment porewater (200–250 µL) was extracted using MicroRhizon filter tubes (Rhizosphere Research Products, Wageningen, Netherlands; 0.2 µm pore size; 0.8 mm diameter) attached to a 1 ml syringe (Torres et al. 2013). The top 10 cm of the sediment were sampled with a vertical resolution of 2.5 mm for the first 2 cm, 5 mm between 2 and 5 cm, and at 10 mm resolution below 5 cm. The porewater samples were immediately transferred to FlipTubes (Semadeni 1.5 ml) and stored on ice in the dark. Samples from each location were analyzed individually for NO_3_^−^, NO_2_^−^, and NH_4_^+^ with two ion chromatography devices, [cations: 882 Compact IC plus, anions: 881 Compact IC pro, Metrohm, Switzerland, with limits of quantification of 2 µmol L^−1^ (NO_3_^−^), 5 µmol L^−1^ (NH_4_^+^), and 0.5 µmol L^−1^ (NO_2_^−^)]. Samples for the analysis of NH_4_^+^ were acidified with HNO_3_ (dilution factor 2) prior to the measurement. All samples were measured within 24 h after extraction. Vertical porewater O_2_ concentration profiles were measured on-site immediately after core retrieval with an O_2_ optode mounted on an automated micro-manipulator (presens, Germany).

### Net sedimentation

An additional sediment core was taken in Lake Sarnen on March 2017 to determine the net sedimentation rate, as well as the total organic carbon (TOC) and nitrogen (TN) content. The sediment was sectioned (resolution of 0.5 cm in the top 20 cm of sediment, 1 cm up to 100 cm of sediment), freeze-dried, ground in an agate mortar and analyzed for TOC and TN by thermic combustion with a Euro EA 3000 Element Analyzer (Hekatech). Total inorganic carbon (TIC) was measured with a Coulometer (CM5015 UIC). Water content was determined by weight difference of wet and dried samples. Porosity was calculated from the water and TOC contents assuming density of the minerals of 2.65 g cm^−3^ (e.g. Och et al. 2012). Net sedimentation rates were obtained from γ-ray measurements of ^210^Pb and ^137^Cs (Benoit and Rozan 2001). For Lake Baldegg, the net sedimentation rate data, as well as the TOC and TN content of the sediment shown in Fig. SI-3, was previously characterized by Steinsberger et al. (2017).

### Water column chemistry and organic matter export from the photic zone

Water column particle traps were installed in both lakes at stations A (Fig. 1, indicated with a star) from March 2017 until November 2018. Each mooring was equipped with two pairs of cylindrical PVC tubes with inner diameters of 9.2 cm and 13.5 cm at 15 m depth and 3 m above the sediment. The sediment traps were subsampled on a biweekly basis, except for the time between November 2017 and February 2018 when material was collected on monthly intervals. The material from parallel traps was pooled, freeze-dried, weighted and mortared. TOC and TN content was analyzed using an Element Analyzer coupled with an Isotope Ratio Mass Spectrometer (EA-IRMS; EA: vario PYRO cube, elementar; IRMS: Iso Prime, GV instruments).

Water column concentrations of O_2_, NO_3_^−^, NO_2_^−^, NH_4_^+^, TN, as well as dissolved inorganic P, total dissolved P and total P were measured monthly from March 2017 to November 2018 from samples collected at the deepest site in both lakes (near the red star in Fig. 1). Depth intervals in Lake Baldegg were 0 m, 2.5 m, 5 m, 7.5 m, 10 m, 12.5 m, 15 m, 20 m, 30 m, 40 m, and 50 m, and in Lake Sarnen 0 m, 2.5 m, 5 m, 7.5 m, 10 m, 12.5 m, 15 m, 20 m, 30 m, 40 m, 50 m, 60 m, 62 m, and 65 m. Parameters were analyzed with the standard methods explicated above.

### Estimation of N removal rates from porewater measurements

We used a one-dimensional reaction-transport model (Müller et al. 2003) applying diffusion coefficients from Li and Gregory (1974) to calculate porewater fluxes based on the vertical concentration gradients of porewater profiles. Best fits were applied to porewater concentration gradients of O_2_, NO_3_^−^ and NH_4_^+^ and fluxes across the sediment water interface determined.

### Estimation of N removal rates from whole-lake N mass balance

Nitrogen removal rates were determined from annual N mass balances of the whole-lake N budget for the time period between 1986 and 2018 (Lake Baldegg) and 1973 and 2018 (Lake Sarnen) as follows:

1 $$V\frac{d\left[ N \right]}{{dt}} = Q\left[ {N_{in} } \right] + N_{atm} + N_{WWTP} - Q\left[ {N_{out} } \right] - N_{NS} - NRR,$$

where V is the lake volume, Q the annual water discharge, [N_in_] and [N_out_] the annual average concentrations of DIN in the inflows and total N in the outflows, N_atm_ the annual N input via atmospheric deposition, N_WWTP_ the N input from waste water treatment plants (WWTP), N_NS_ net sedimentation and NRR the amount of annually transformed N.

### Estimation of N removal rates from hypolimnion N mass balances

Mass balances of TN in the annually stratified hypolimnia for the years 2017 and 2018 were established based on our data from the water column monitoring, sediment traps and porewater fluxes to quantify the NRR:

2 $$V_{hypo} \frac{{d\left[ {TN} \right]}}{dt} = N_{NEP} + N_{NIT} - N_{GS} - NRR,$$

where V_hypo_ is the hypolimnion volume (below 15 m depth), N_NEP_ is the net ecosystem production of particulate N from the epilimnion determined from the load and composition of settling particles from the sediment traps at 15 m depth. N_NIT_ is the nitrification of NH_4_^+^ diffusing from the sediment (estimated from porewater concentration profiles) and N_GS_ is the gross sedimentation determined from the sediment trap 3 m above sediment. The change of the hypolimnion’s TN content between spring and autumn, $V_{hypo} \frac{{d\left[ {TN} \right]}}{dt}$, was calculated from water column measurements and lake bathimetry. Contrary to Lake Baldegg, Lake Sarnen was not monitored continuously by the responsible authorities. Chemical water profiles for N and P parameters in spring and fall were only available for 14 out of 47 years. The N content in spring of missing years were interpolated in order to estimate NRR.

### Load estimates of dissolved nitrogen from historical monitoring data

The load of dissolved inorganic nitrogen (DIN) to Lake Baldegg is monitored by the Cantonal Bureau for the Environment Lucerne since 1986, not including particulate and dissolved organic N input. Concentrations of NO_3_^−^ and NH_4_^+^ were monitored on 5 out of 21 tributaries (37% of the catchment) and upscaled to the full catchment area. Samples were collected regularly every 3 weeks, with additional samples during floodwater events triggered automatically when water discharge monitoring exceeded a threshold value. Contributions to the N load from the local sewage treatment plant, storm-water overflow, and precipitation were also included. The total water discharge from Lake Baldegg required to calculate export loads was recorded using automated level gauges.

In Lake Sarnen, the water column was monitored for NO_3_^−^, NO_2_^−^, and NH_4_^+^ since 1972. Sampling campaigns were discontinuous and datasets of only 14 years were available between 1972 and 2016. Volume-weighted average concentrations of DIN after winter mixing of years without monitoring missing years were linearly interpolated to serve as estimates for annual budget calculations. Tributary monitoring data was not available for the Lake Sarnen catchment. Instead, the N load was estimated applying the MODIFFUS model, which has been widely applied and calibrated in Switzerland (Hürdler et al. 2015). The model calculates the N load to the lake according to catchment soil type, land use, agricultural practices, ground slopes and precipitation (Table SI-1). Due to the large catchment-to-lake area ratio, Lake Sarnen had a greater per area N load estimate than the more eutrophic Lake Baldegg. This is consistent with the observation that Lake Sarnen received a higher fraction of allochthonous and particulate matter input compared to Lake Baldegg. In Lake Sarnen, the export loads of total nitrogen (TN) were calculated from monthly water column monitoring data (March 2017 to November 2018) and daily average hydraulic loads recorded by the Swiss Federal Office for the Environment (FOEN 2020). TN concentrations from samples collected at 0.5 m and 2.5 m water depth were averaged, and then multiplied by the total monthly water discharge.

Statistical tests of correlations significance were performed by Pearson’s correlation coefficient.

## Results

### Contrasting carbon and nitrogen sedimentation regimes in Lakes Baldegg and Sarnen

While in Lake Baldegg, deposition rates of organic C and N were dominated by the large spring phytoplankton blooms (April–June, Fig. SI-1a), productivity in Lake Sarnen was low and its sedimentation regime was strongly shaped by the hydrology of its tributaries (Fig. SI-1b). Also the elemental C:N ratio characterizes Lake Baldegg as autochthonously dominated, while Lake Sarnen was often exposed to stormwater events causing turbidity currents with allochthonous material permeating the epilimnion and dispersing in the hypolimnion. The settling organic matter of Lake Baldegg was rich in N relative to C, for instance, the spring–summer period coincided with a minimum in the C:N ratio averaging 4.8 (Fig. SI-1g), which was lower than the Redfieldean C:N stoichiometry of 6.6. In contrast, the high C:N ratios in Lake Sarnen (Fig. SI-1h) were indicative of allochthonous organic matter. Table 2 summarizes the deposition rates obtained from the sediment traps exposed in both lakes. (A detailed description is given in chapter SI-1).

**Table 2:** Results from sediment traps averaged for 2017 and 2018 in Lakes Baldegg (BAL) and Sarnen (SAR)

Parameter	Dim	BAL		SAR	
		Upper	Lower	Upper	Lower
C:N ratio	mol/mol	5.9 ± 1.7		8.5 ± 2.4	
Bulk depos. rate	g m^−2^ year^−1^	1470	2320	–	–
TOC depos. rate	gC m^−2^ year^−1^	56.8	90.9	28.6	–
TN depos. rate	gN m^−2^ year^−1^	11.2	16.8	4.1	–
TN depos. (Apr–Oct)	gN m^−2^	7.7	9.6	2.5	3.1
N net sed. (dated cores)	gN m^−2^ year^−1^		4.9		6.9

Data from Lake Sarnen in 2017 were biased by a large storm surge, thus we rely on the data collected from April to October, 2018. N net sedimentation is based on dated sediment core analysis (shown in Fig. SI-3)

Overall, the 2017–2018 sedimentation rate, according to the upper trap data, was roughly twofold higher in Lake Baldegg (56.8 g C m^−2^ year^−1^ and 11.2 g N m^−2^ year^−1^) than in Lake Sarnen (28.6 g C m^−2^ year^−1^ and 4.1 g N m^−2^ year^−1^; Table 2). Notably, the estimate for Lake Sarnen represents the upper limit due to the increased sedimentation rates induced during the winter overturn period and as a consequence of the 2017 storm event. Furthermore, the sinking organic matter in Lake Baldegg (C:N ratio of 5.9 ± 1.7) was more enriched in N compared to Lake Sarnen (C:N ratio of 8.5 ± 2.4; Table 2, Fig. SI-1g, h). Sediment core analysis of Lake Baldegg also revealed two- to threefold higher TOC and TN content in the upper sediments compared to Lake Sarnen (Fig. SI-1). The estimated net N sedimentation rate in Lake Baldegg was 4.9 gN m^−2^ year^−1^, which was over twofold lower than the export from the epilimnion (11.2 gN m^−2^ year^−1^; Table 2), suggesting that the sediments support active turnover of organic N. While in Lake Sarnen, the N burial (6.9 gN m^−2^ year^−1^) was higher than the N input to the sediments (4.1 gN m^−2^ year^−1^), frequent storm water events like the one reported in 2017, could account for the enhanced sedimentary N loading regime. Indeed, the Lake Sarnen catchment area is threefold greater than Lake Baldegg, and is thus likely to be more heavily influenced by the tributary hydrology and the input of allochthonous matter.

### Porewater fluxes of oxygen, nitrate and ammonium across the sediment–water interface

The high organic matter content in Lake Baldegg sediments contributed to significant O_2_ respiration, which constrained O_2_ penetration to less than 4.5 mm depth (Fig. SI-4c). While in the more organic matter poor Lake Sarnen sediments, O_2_ penetration was deeper at 3–18.5 mm (Fig. SI-4d). Over our seasonal campaign in Lakes Sarnen and Baldegg, we find that the O_2_ penetration depth, as well as the O_2_ concentrations immediately above the sediment–water interface, varied significantly from 0.1 to 10.3 mg L^−1^ and 4.8 to 11.2 mg L^−1^, respectively (Fig. SI-4a, b). Over the course of the seasonal campaign, we find that bottom water O_2_ concentrations were greatest after the winter overturn period and early summer (between 9 to 11 mg L^−1^ Fig. SI-4a, b), which also corresponded with the largest O_2_ penetration depth into the sediments (Fig. SI-4c, d). Furthermore, in Lake Baldegg, the highest O_2_ flux into the sediments occurred in spring, which reached a maximum of 14.8 mmol m^−2^ day^−1^.

**Fig. 2:**
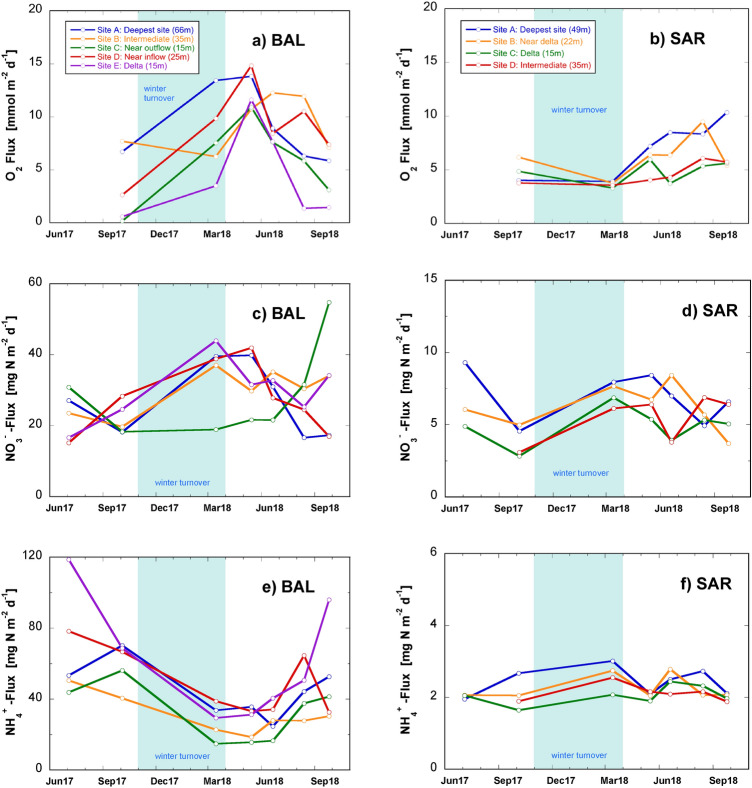
Nutrient (NO_3_^−^ and NH_4_^+^) and O_2_ fluxes measured across the water–sediment interface. Porewater analyses were performed on recovered cores at all sampling stations indicated in Fig. 1. Fluxes of NH_4_^+^ were directed from the sediment towards the bottom water and fluxes of NO_3_^−^ were directed from the water into the sediment. Please note the different scale for the two lakes. The shaded area depicts the period of winter overturn

In contrast to BAL, where O_2_ concentrations in the sediment-overlying water could drop to < 1 mg L^−1^ in spite of the artificial aeration, they were always > 6.8 mg L^−1^ in the bottom waters of Lake Sarnen (Fig. SI-4b). The O_2_ flux was lowest in early spring reaching 3.9 mmol m^−2^ day^−1^. In late summer, both lakes experienced their lowest O_2_ concentrations in the bottom waters and the O_2_ penetration depth was significantly reduced by as much as 75% compared to the spring period. In Lake Baldegg, for instance, the O_2_ concentrations decreased to a low of < 1 mg L^−1^ towards the end of the stratified season due to the accumulation of surface exported organic matter in sediments. While there was some degree of spatial variability between the magnitudes of the O_2_ fluxes between different sites, most sites held a consistent seasonal pattern (Fig. 2a, b).

In Lake Baldegg, the fluxes of NO_3_^−^ from the water to the sediment were in the range of 15–55 mg N m^−2^ day^−1^ (average of 10.4 ± 3.4 g N m^−2^ year^−1^) (Fig. 2c) while NO_3_^−^ concentrations in the water overlying the sediment was always in the range of 1.2–1.4 mg N L^−1^ without seasonal fluctuations (Fig. SI-5). At most sites, the NO_3_^−^ flux was highest in early spring (~ 40 mg N m^−2^ day^−1^) following the winter overturn period, which then decreased by half by the end of the stratified period in late summer (~ 20 mg N m^−2^ day^−1^). Site C, located near the lake outflow, was the notable exception to this trend (green line in Fig. 2c), otherwise we observed no notable systematic differences between the various sites. In the oligotrophic Lake Sarnen, NO_3_^−^ fluxes were about 5 times smaller than in Lake Baldegg ranging from 3 to 9 mg N m^−2^ day^−1^ (average of 2.2 ± 0.6 g N m^−2^ year^−1^). Similar to Lake Baldegg, the NO_3_^−^ fluxes at the sediment approximately doubled between autumn and the following spring. Values obtained at different locations did not show systematic divergence (Fig. 2d).

Fluxes of NH_4_^+^ from the Lake Baldegg sediments increased in average from 28 to 45 g N m^−2^ year^−1^ towards the end of the stratified period as a consequence of the accumulation and mineralization of the freshly deposited organic matter during the productive season (Fig. 2e). During the non-stratified period from November to March, NH_4_^+^ fluxes dropped again by 40–75%. In Lake Sarnen, the deposition of organic matter in this very oligotrophic lake was small enough to prevent accumulation of NH_4_^+^ in the bottom waters. Porewater fluxes of NH_4_^+^ were 2.2 ± 0.3 g N m^−2^ year^−1^ (average of 0.8 ± 0.1 g N m^−2^ year^−1^) without a pronounced seasonality (Fig. 2f).

In summary, despite the lack of spatial differences across the lakes, we find strong seasonal patterns in the porewater fluxes of Lake Baldegg, but much less pronounced in Lake Sarnen. For instance, the highest NRR, based on NO_3_^−^ fluxes, occurred in early spring and decreased in late autumn. The peak NRR appeared to coincide with high bottom water O_2_ concentrations (Fig. SI-4a, b).

### Quantification of N removal rates (NRR) based on whole lake budgets

**Fig. 3:**
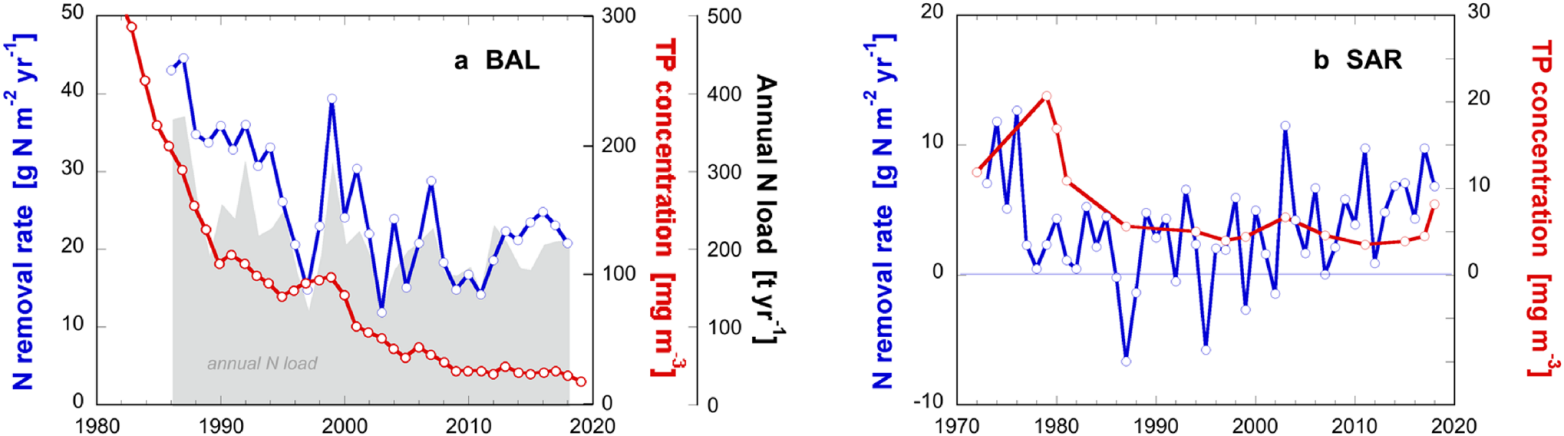
Annual nitrogen removal rates (blue) estimated from whole-lake budgets plotted also in relation to the concentration of TP (red). **a** Lake Baldegg (1986–2018), and **b** Lake Sarnen (1972–2018). The red lines signify the concentration of TP after winter turnover. The gray shaded area in **a** depicts the annual areal load of dissolved N (color figure online)

We analyzed whole lake N budgets using long-term monitoring data from Lakes Baldegg (1985–2018) and Sarnen (1974–2018) to estimate removal rates of dissolved N (which includes denitrification, DNRA and anammox) according to Eq. (1) (Fig. 3). A decreasing trend in the N removal was observed in Lake Baldegg since the start of the measurements in 1985 until the mid-1990s (Fig. 3a) with an average of 22 ± 6.0 g N m^−2^ year^−1^ over the past 24 years. A similar decrease of the annual average load of dissolved inorganic N was also apparent with an average of 197 ± 40 t N year^−1^ for the past 24 years.

In Lake Sarnen, due to the limited amount of monitoring data, the large variations of the lake’s N content and the relatively small N fraction removed, resulted in low and sometimes negative removal rates. However, the long dataset reaching back to 1972 ensured a reliable range of 3.2 ± 4.2 g N m^−2^ year^−1^ in Lake Sarnen (Fig. 3b), which is ~ 6 times lower than for Lake Baldegg. The total N load to Lake Sarnen was estimated to 223 t N year^−1^ applying the catchment model MODIFFUS (Hürdler et al. 2015).

### Nitrogen removal rates estimated from seasonal hypolimnion budgets

**Fig. 4:**
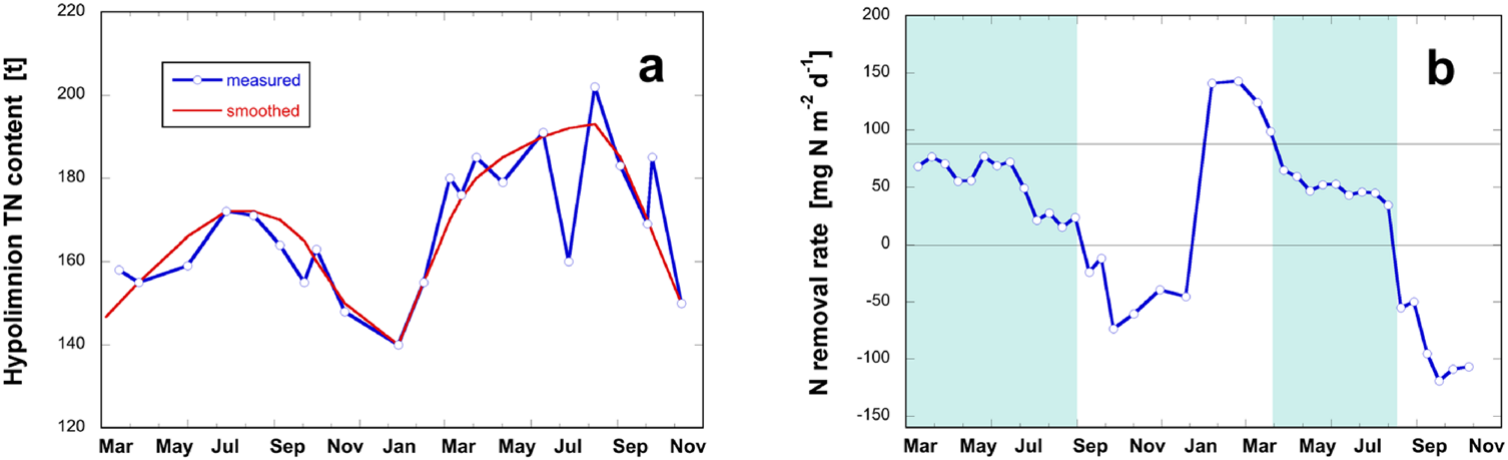
Estimation of N removal rates based on the hypolimnion budget of Lake Baldegg (2017–2018). **a** Hypolimnion content of TN: estimated from measurements in blue, smoothed curve in red. **b** Bi-weekly estimation of N removal rates during summer stratification (shaded) (color figure online)

For Lake Baldegg, we established hypolimnion budgets during the stratified periods in 2017 and 2018 to estimate the N removal according to Eq. (2). Reproducibility and precision of analytical TN determination were crucial for the calculation of monthly gradients in the lake’s water column. The temporal succession of estimated TN contents of Lake Baldegg (Fig. 4a) required smoothing (red line) to reduce the variability of the subsequently calculated removal rates (Fig. 4b). The N removal was relatively constant during the stratified period in both years (shaded) with a mean rate of 21 ± 4.6 g N m^−2^ year^−1^.

It was not possible to establish a sufficiently accurate TN-hypolimnion budget for Lake Sarnen because of the small changes in the TN content of the hypolimnion. Monthly differences of the TN content were smaller than the margins of analytical reproducibility and precision of TN measurements in the water column, leaving it impossible to calculate removal rates.

### Annual nitrogen removal rates from porewater measurements and whole-lake budgets

Table 3 summarizes results from all three ways of estimating removal of dissolved N. In the eutrophic Lake Baldegg with its high NO_3_^−^ concentration and long water residence time, N was mainly removed (53%) via sediment diagenetic processes such as denitrification, DNRA, and anammox, whereas in the oligotrophic Lake Sarnen with almost threefold less NO_3_^−^ and five times shorter water residence time these processes contributed only 10%, and sediment burial was the main pathway for N removal (22%). The export via outflow in Lake Baldegg was only 22%, while 77% of the load left Lake Sarnen unaffected. The total areal N removal efficiency (NRE, diagenetic processes plus net sedimentation) was 2.5 times higher in the eutrophic than in the oligotrophic lake.

**Table 3:** Nitrogen removal rates in Lakes Baldegg (BAL) and Sarnen (SAR) based on porewater measurements, whole-lake budgets, and hypolimnion budgets

Method	BAL	SAR
	g N m^−2^ year^−1^	
Total areal load	38 ± 8 (100%)	31 (100%)
N removal rates (NRR) estimated from		
Porewater measurements	10.4 ± 3.4	2.2 ± 0.6
Whole-lake budgets	20 ± 6.6 (53%)	3.2 ± 4.2 (10%)
Hypolimnion budget (2017–2018)	21 ± 4.6	–
N net sedimentation	4.9 (13%)	6.9 (22%)
Export by outflow (2017–2018)	8.3 (22%)	24 (77%)
N removal efficiency (NRE)	24.9 (66%)	10.1 (33%)

Percent N-removal was calculated with results for the whole-lake N-budget only. The sum of all contributions is ≠ 100% because all parameters were measured independently, and the total areal load was set to 100*%*

Estimates of N removal rates from NO_3_^−^ porewater profiles were clearly lower than the rates determined with budget calculations. This suggests that some N removal processes escape the quantification by porewater NO_3_^−^ profiling (such as e.g. combined nitrification–denitrification) or occur in locations other than the sediment surface layer (e.g., in the metalimnic oxygen minimum zone, in sinking organic particles or the littoral sediment). This discrepancy is more expressed in the eutrophic Lake Baldegg than in the oligotrophic lake Sarnen.

## Discussion

### Nitrogen removal rates from two lakes of contrasting trophic levels

Lake Baldegg is a nutrient loaded and organic matter-rich lake with an artificially aerated hypolimnion to prevent bottom water hypoxia. It is contrasted with Lake Sarnen, which lies in a mountainous catchment that experiences lower external nutrient loading and primary productivity. Our results find that the N removal rates (NRR) are vastly different between Lake Baldegg (20 ± 6.6 g N m^−2^ year^−1^) and Lake Sarnen (3.2 ± 4.2 g N m^−2^ year^−1^) (Table 3). These NRRs are within the range reported for other oligotrophic lakes of 0.5–3.9 g N m^−2^ year^−1^, and of other eutrophic lakes of 3.9–9.2 g N m^−2^ year^−1^; with exceptional cases reaching up to 38 g N m^−2^ year^−1^ (Hasegawa and Okino 2004; Heinen and McManus 2004; Müller et al. 2005; Pina-Ochoa and Alvarez-Cobelas 2006; McCrackin and Elser 2010; Bruesewitz et al. 2012; Li and Katsev 2014).

Despite the difference in the NRR between Lake Baldegg and Lake Sarnen, both lakes sustain active N turnover in the upper sediment layer. In Lakes Baldegg and Sarnen both sediments serve as sinks of O_2_ and NO_3_^−^, and sources of NH_4_^+^ (Fig. 2). They observed an annual maximum in the NRR at the beginning of stratification, specifically between the high organic matter rain rate over the mixing season and the spring phytoplankton bloom.

In Lake Sarnen, the low concentration of TP (~ 5 mg P m^−3^) greatly constrains the intensity of the phytoplankton bloom, and therefore restricts the export rates of organic matter. This likely contributes to the overall lower NRR in Lake Sarnen compared to Lake Baldegg (Fig. 3). Furthermore, the high N- and P-loading in Lake Baldegg not only sustains larger organic matter export rates, but the organic matter delivered to the sediments is far more N-rich relative to carbon (C:N of 6.0 ± 1.6), than the settling organic matter stoichiometry observed in Lake Sarnen (C:N of 7.9 ± 0.7) (Fig. SI-1g, h). Previous studies have also highlighted that phytoplankton grown under NO_3_^−^ and P replete conditions assimilate N and P at faster rate relative to C, other factors such as temperature and light also moderate algal biomass C:N stoichiometry (Healey and Hendzell 1976; Moreno and Martiny 2018). We suggest that external N- and P-loading controls both the rates of organic matter export and its N-richness, which has important implications for NRR in underlying sediments.

### Load-dependent nitrogen removal rate in Lake Baldegg

**Fig. 5:**
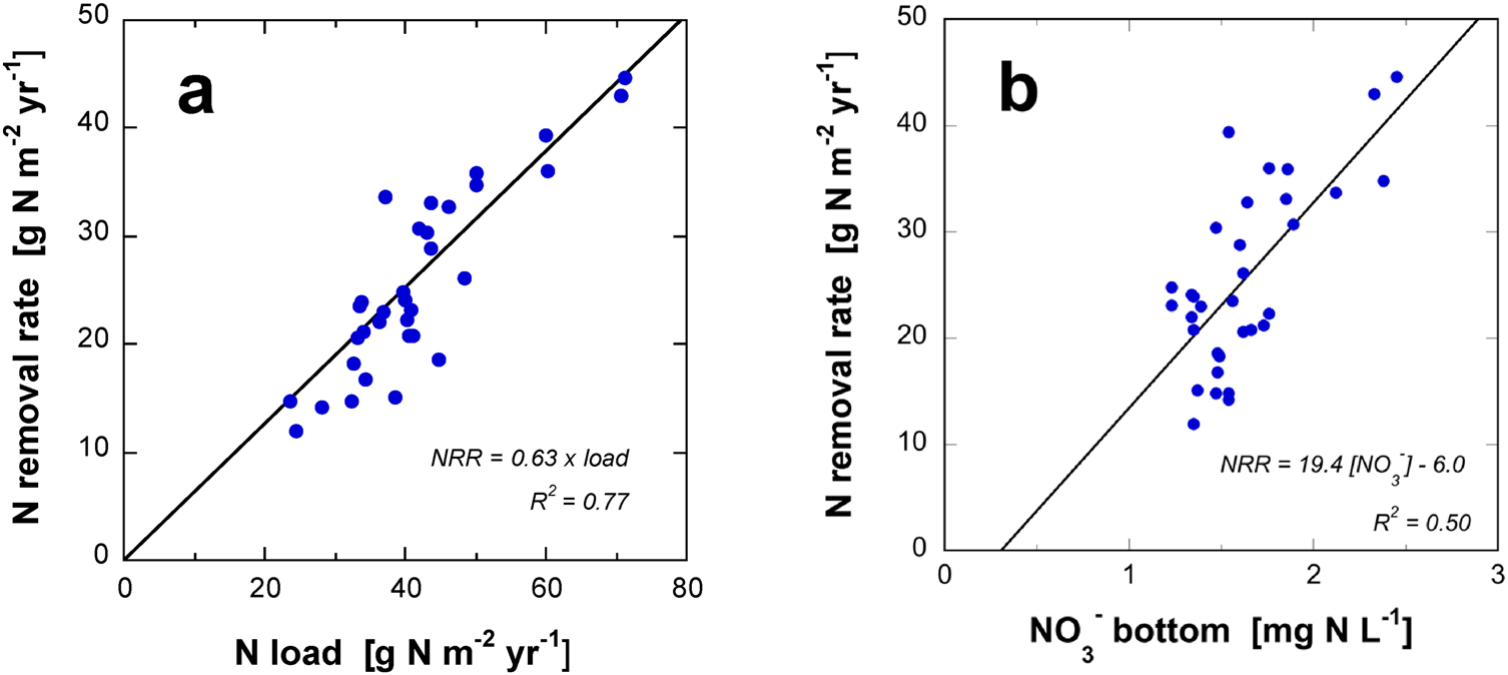
Increasing N removal rates (NRR) with **a** increasing annual load of dissolved N, and **b** increasing NO_3_^−^ concentrations in the bottom water of Lake Baldegg (averages of April–October, 1986–2019, deepest location). The linear regression in **a** with a slope of 0.63 was forced through the origin (p value of both correlations < 0.00001)

Analyzing the 33-years monitoring dataset from Lake Baldegg, a strong correlation of NRR with annual loads of dissolved N was observed. The NRR was proportional to the loading rate of dissolved N with a multiplication factor of 0.63 (Fig. 5a). The difference to Seitzinger et al. (2006) who reported a proportionality factor of 0.26 may be owed to the fact that they used total N inputs while we had data on DIN loads available.

NRR was also related to the average bottom water concentrations of NO_3_^−^ (April–October, deepest sampling depths, R^2^ = 0.50, Fig. 5b) indicating a direct link to denitrification, which is controlled by the diffusion of NO_3_^−^ across the benthic boundary layer and into the sediment and must thus be proportionately related to its concentration above the sediment–water interface (Pina-Ochoa and Alvarez-Cobelas 2006). However, NO_3_^−^ concentrations in the deep water require a full mixing period to equilibrate with the input during the past stratified season and therefore, the NRR determined from an annual balance would only apply in a steady-state situation where N-loads, water, etc. remained the same. Therefore, the good correlation of NRRs with annual loads implies that the imported N (or, a fraction proportional to it) has an immediate effect on N removal, i.e. it is transferred to the hypolimnetic sediment or reacts within the epilimnion with little temporal delay. Water masses from tributaries during the stratified season mix in the lakes’ epilimnion, and transfer of N to the hypolimnion occurs only by assimilation and sedimentation. Only lakes where primary production is controlled by N could immediately react on changes of the N load in the same year. This can be excluded in Lake Baldegg with its high concentration of NO_3_^−^ that was never exhausted in the productive zone, because the lake was fully productive since more than a century (Lotter 1998) with P concentration not limiting production. Indicative for the situation is the hypolimnetic O_2_ consumption that is unchanged since measurements begun in 1983 even though TP concentration decreased from 520 to 25 mg m^−3^ (Müller et al. 2019), and net ecosystem production of ~ 90 g C m^−2^ year^−1^ both in 1995/1996 (Müller et al. 2012) and 2013/2014 (Steinsberger et al. 2017) determined from sediment traps. Hence, the good correlation between NRR and load requires that a large fraction of the imported N is removed within the epilimnion, e.g. within the epilimnic minimum zone (Yu et al. 2014; Kogo et al. 2013; Grantz et al. 2012; Serruya 1972), in the warmer littoral sediment (Palacin-Lizarbe et al. 2020; Rissanen et al. 2011; Saunders and Kalff 2001b), or in anoxic microzones of settling particulate organic matter (Stief et al. 2016; Bianchi et al. 2018; Xia et al. 2017; Liu et al. 2013; Michotey and Bonin 1997; Hietanen 1998; Grossart and Simon 1993). The substantial difference in the estimation of NRR from NO_3_^−^ porewater concentration gradients and whole lake budgets (Table 3) as well indicate that a fraction of N removal does not occur at the sediment surface and thus goes untraced by porewater measurements. This subject requires further investigation in future surveys.

It has been hypothesized that mitigation of P pollution of surface waters affects the N removal capacity of lakes due to reduced assimilation into algal biomass and the decline of reducing conditions in lakes (Finley et al. 2013; Bernhardt 2013). Indeed, a simultaneous decrease of the concentrations of TP and DIN in Lake Baldegg occurred, at least in the first years after the beginning of measurements (Fig. 3). However, as mentioned above, we have no indications of a decreasing primary productivity since the hypolimnetic O_2_ consumption and the net ecosystem production remained unchanged during the past decades. We think therefore that the simultaneous decrease of TP concentration and NRR were parallel trends that were not related. Both loads of TP and N decreased due to the elimination of household sewage disposal and mitigation measures in agricultural practices. In addition, the 35 years of artificial oxygenation of the lake helped to sustain an oxic sediment surface, independent of the primary production of the lake.

### Seasonally increased O_2_ concentrations enhance N removal rates

Aside from N and P, our findings further indicate a strong role for O_2_ in influencing NRR. Previous studies were not specifically focused on the impact of bottom water O_2_ as a possible control of denitrification (Wall et al. 2005; Alvarez Cobelas et al. 2019), although Rissanen et al. (2013) mention the possibility that the extension of the oxic nitrification zone in the sediment may enhance denitrification via increased nitrate concentration (Rysgaard et al. 1994). Also in Lake Baldegg, a previous report has inferred that increasing O_2_ ventilation in bottom waters might augment denitrification in shallow sediments (Mengis et al. 1997). The idea that O_2_ could stimulate denitrification is counter to the long-held notion that denitrification is a strictly anaerobic process, and is only favored when O_2_ concentrations subside below a distinct threshold (Seitzinger et al. 2006). A growing number of studies have indeed shown that denitrification can thrive under oxic conditions in permeable marine sediments (Gao et al. 2010; Ji et al. 2015; Marchant et al. 2016). In more O_2_ permeable sediments ^15^N tracer studies have found that nitrification–denitrification can play an important role in N-loss in eutrophic sediments, particularly also if NO_3_^−^ concentrations are maintained at low levels (Marchant et al. 2016; Rysgaard et al. 1993 and references therein).

**Fig. 6:**
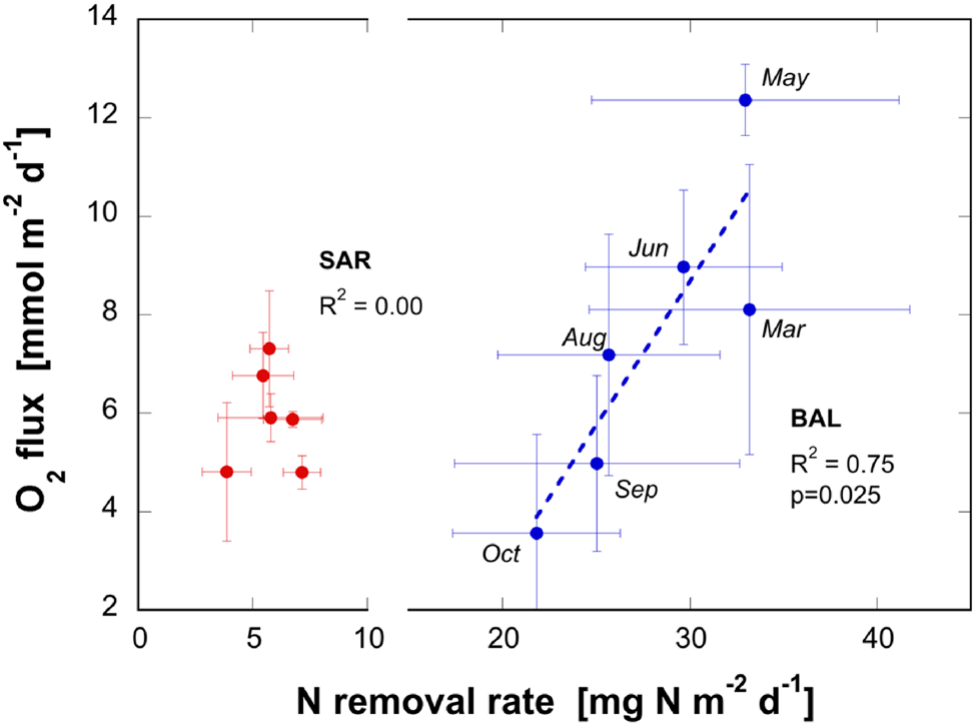
Correlation between N removal rates (from porewater measurements, Fig. 2c, d) and O_2_ fluxes in the sediment overlying water in Lakes Baldegg (blue) and Sarnen (red). N removal rates and O_2_ concentrations were averaged for each sampling date over all sites measured. It is a trend over seasonal data. The correlation in Lake Baldegg was significant at p = 0.025 (color figure online)

Our seasonal sampling campaign in 2018 in Lake Baldegg finds that the greatest rates of NRR occured in early spring, shortly following the winter overturn when O_2_ concentrations were highest in bottom waters (Fig. SI-4a, b). This period is demarcated by much deeper O_2_ penetration into the upper sediments, in both Lakes Baldegg and Sarnen (Fig. SI-4c, d). Plotting the seasonal data, we find a clear relationship between O_2_ fluxes and NRR in the sediments of the eutrophic Lake Baldegg (R^2^ = 0.75, p = 0.025), whereby NRR increased as a function of increasing O_2_ fluxes (Fig. 6). NO_3_^−^ concentrations in the sediment-overlying water remained between 1.2 and 1.4 mg N L^−1^ (Fig. SI-5a) and although denitrifiers depend on the concentration of NO_3_^−^ as an essential substrate, no significant effect on NRR was apparent due to the small concentration range covered. Furthermore, the increased concentration of O_2_ in early spring also coincided with a peak in the rates of organic matter deposition (Fig. SI-1). The higher O_2_ availability may stimulate breakdown of organic matter and thus again provide more organic substrate for denitrifiers. The present dataset does not allow estimating its contribution to the increase of NRR. However, as Lake Baldegg is fully productive and the sediment rich in organic matter, it is unlikely that this was a significant cause for the spring increase of NRR even though it may add to it. Contrary to Lake Baldegg, no correlation between the fluxes of O_2_ and NRR were observed in Lake Sarnen due to the low productivity and thus low release of NH_4_^+^.

Given that O_2_ is a strong oxidant of organic matter, we hypothesize that the high O_2_ flux at the beginning of spring mediates enhanced organic matter respiration of freshly settled material, generating remineralized NH_4_^+^. Remineralized NH_4_^+^, in combination with the elevated O_2_ flux to the upper sediments ostensibly promotes nitrification, which controls NO_3_^−^ availability for denitrification. Indeed, stable isotope ^15^N tracer experiments, performed on sediments recovered from Lakes Baldegg and Sarnen, find evidence of a tight nitrification–denitrification coupling in the oxic–anoxic transition zone (Callbeck et al., in prep). Moreover, nitrification–denitrification co-occurred with high rates of organic N mineralization in the narrow upper boundary layer. In this study, we therefore suggest that O_2_ indirectly moderates the availability of NO_3_^−^ supporting denitrification, which appears to be most relevant in early spring following the winter turnover period and at the onset of the spring bloom. In Lake Sarnen, the porewater flux of NH_4_^+^ is ~ 20 times lower than in Lake Baldegg (Fig. 2e, f) and thus, the effect of increased NO_3_^−^ availability due to nitrification may not be visible (red marks in Fig. 6).

Direct coupling of nitrification–denitrification could contribute to the higher NRR estimated from whole-lake budgets than from sediment porewater NO_3_^−^ gradients (Table 3). This process would not be detectable from porewater concentration gradients. As Lake Baldegg has a long eutrophic history over the past 140 years, and a rich legacy of organic matter buried in its sediments (Steinsberger et al. 2017), fluxes of NH_4_^+^ from the sediment are still high. In combination with the artificial aeration assuring high concentrations of O_2_ at the sediment–water interface this may stimulate an exceptionally high rate of combined nitrification–denitrification activity.

### Nitrogen removal efficiency (NRE)

Both Lakes Baldegg and Sarnen also observed striking differences in the NRE, which is defined as the proportion of N input removed via transformation to N_2_ and permanent burial. Lake Baldegg removed two thirds of the load of dissolved N, while Lake Sarnen eliminated one third (Table 3). Previous global lake surveys have shown that water residence time is strongly correlated with NRE, whereby the longer the water residence time the more effective the lake is at removing the input of N (Finlay et al. 2013; Tong et al. 2019). In line with this, the fivefold longer water residence time of Lake Baldegg compared to Lake Sarnen likely contributes to its 2.5 times higher per-area-NRE (Table 3).

A low NRE has the potential to exert greater pressure on downstream ecosystems to remove N. Despite the oligotrophic state of Lake Sarnen, because it removes very little of the external N load, the N outflow rate is threefold greater than of the eutrophic Lake Baldegg. When placed in context of the large catchment area, Lake Baldegg serves as a critically important “natural” filter of N thereby mitigating downstream eutrophication in other sensitive ecosystems.

## Conclusions

Estimation of NRR by sediment porewater measurements of NO_3_^−^ proved feasible and a less laborious and time consuming alternative to N-budgeting methods. Negligible differences between various lake sites but pronounced seasonality was observed. The smaller estimates of NRR from porewater fluxes compared to N-budgets suggest that the sediment surface may not exclusively be the location of N removal processes, and/or that combined nitrification-denitrifcation plays a role, which would escape detection by porewater measurements. From the correlation of bottom water O_2_ concentrations and NRR, we hypothesize that O_2_ stimulated mineralization of settled organic matter and may have enhanced combined nitrification–denitrification, which requires further investigation.

## Supplementary Information

Below is the link to the electronic supplementary material.

Supplementary file1 (DOCX 5467 KB)
