# Supplementary material for: Nitrogen removal processes in lakes of different trophic states from on-site measurements and historic data
Source: Aquat Sci. 2021 Mar 10;83(2):37. doi: 10.1007/s00027-021-00795-7 (PMC7946664; doi:10.1007/s00027-021-00795-7)
Supplement: Supplementary file 1 — Supplementary file1 (DOCX 5467 KB) [file 27_2021_795_MOESM1_ESM.docx]

## Supporting Information File for

## Nitrogen removal processes in lakes of different trophic states from on-site measurements and historic data

Beat Müller*, Raoul Thoma, Kathrin B. L. Baumann, Cameron M. Callbeck, Carsten J. Schubert

Eawag, Swiss Federal Institute of Aquatic Science and Technology,
CH-6047 Kastanienbaum, Switzerland

* Corresponding Author: Beat Müller, e-mail :beat.mueller@eawag.ch

January, 2021

## Chapter SI-1: Contrasting carbon and nitrogen sedimentation regimes in Lakes Baldegg and Sarnen

In Lake Baldegg, deposition rates of organic C and N were dominated by the large spring phytoplankton blooms (April to June) and the smaller summer blooms from July to September (Fig. SI-1a, c, e). The peak bloom periods induced an increase in the C and N sedimentation rates in both the upper and lower traps reaching up to 300 mg C m^-2^ d^-1^ and 75 mg N m^-2^ d^-1^. The mean organic C and N export rates to the sediment for the two spring-summer periods equated to 43 mg C m^-2^ d^-1^ and 10 mg N m^-2^ d^-1^. Moreover, the settling organic matter was rich in N relative to C, for instance, the spring-summer period coincided with a minimum in the C:N ratio averaging 4.8 (Fig. SI-1g), which was lower than the Redfieldean C:N stoichiometry of 6.6.

During the winter mixing period (November to March, shaded areas in Fig. SI-1), both the upper and lower sediment traps reported a large secondary increase in the C and N sedimentation rates up to 410 mg C m^-2^ d^-1^ and 58 mg N m^-2^ d^-1^; although the lower trap reported a two times higher export rate than the upper trap (Fig. SI-1c, e). Sediment resuspension likely explains the strong secondary maximum observed in winter, and the larger accumulation of organic material reported in the lower sediment trap is likely due to sediment focusing (Steinsberger et al. 2017). Furthermore, the settling particles during the winter mixing period had a mean C:N ratio of 8.0, which was relatively N-poor in comparison to the summer period (Fig. SI-1g). This supports the notion that the particles collected in winter consisted partly of already degraded material.

While the origin of organic matter in Lake Baldegg was almost entirely autochthonous, the sedimentation regime of Lake Sarnen was strongly shaped by the hydrology of its tributaries (Fig. SI-1b). Storm-water events (e.g. of May and December 2017) caused turbidity currents with allochthonous matter permeating the epilimnion and dispersing in the hypolimnion, thereby causing massive spikes in the C and N sedimentation rates (Fig. SI-1d, f). The high C:N ratios were indicative of allochthonous organic matter. The upper trap, which was positioned higher in the water column, was naturally less influenced by resuspension and therefore offered a more reliable assessment of seasonal sedimentation patterns in Lake Sarnen. In the 2018 spring-summer period, the upper trap reported a gradual 3- to 5-fold increase in the C and N export rates from June to September, reaching a peak value of 140 mg C m^-2^ d^-1^ and


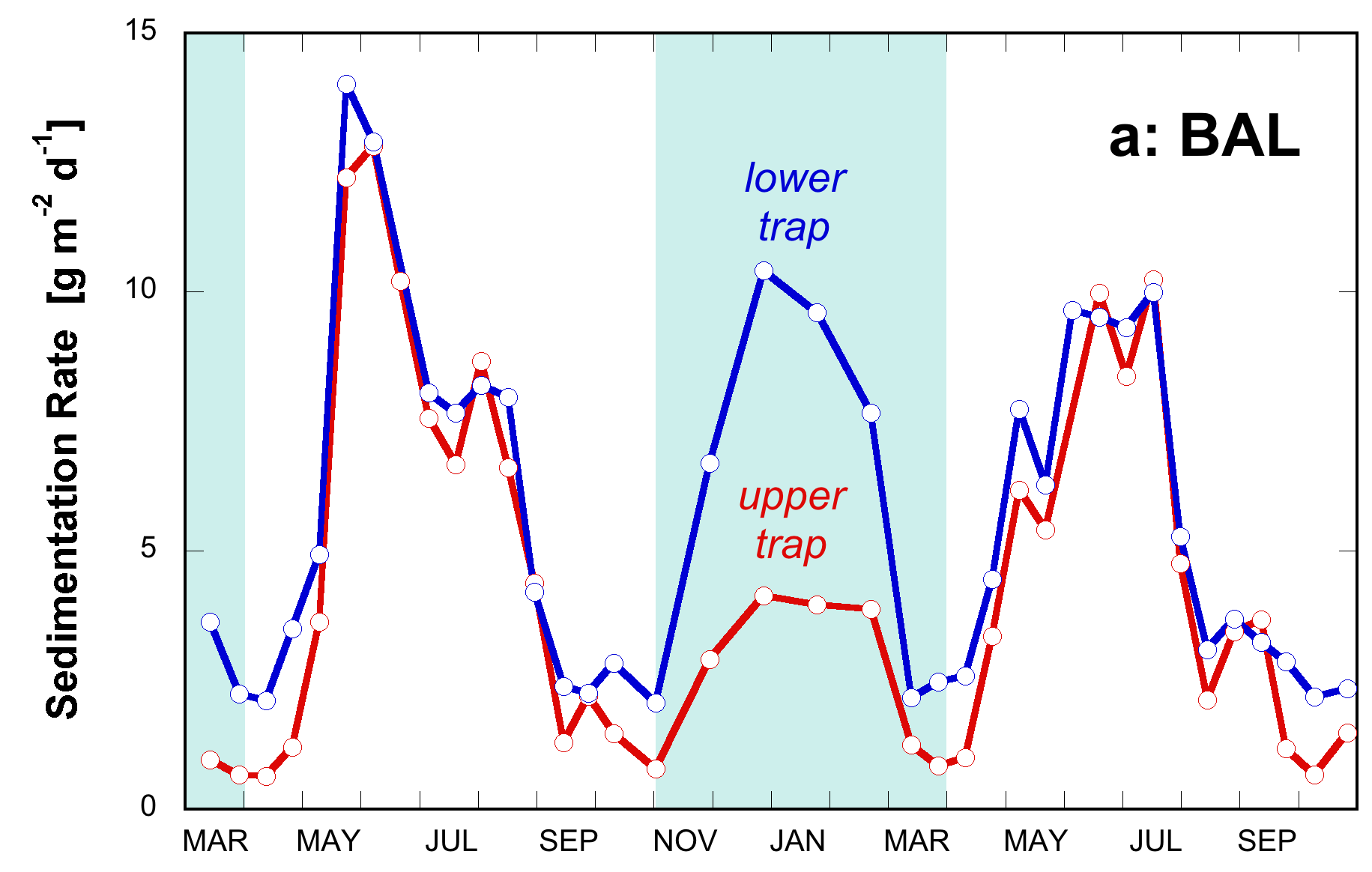

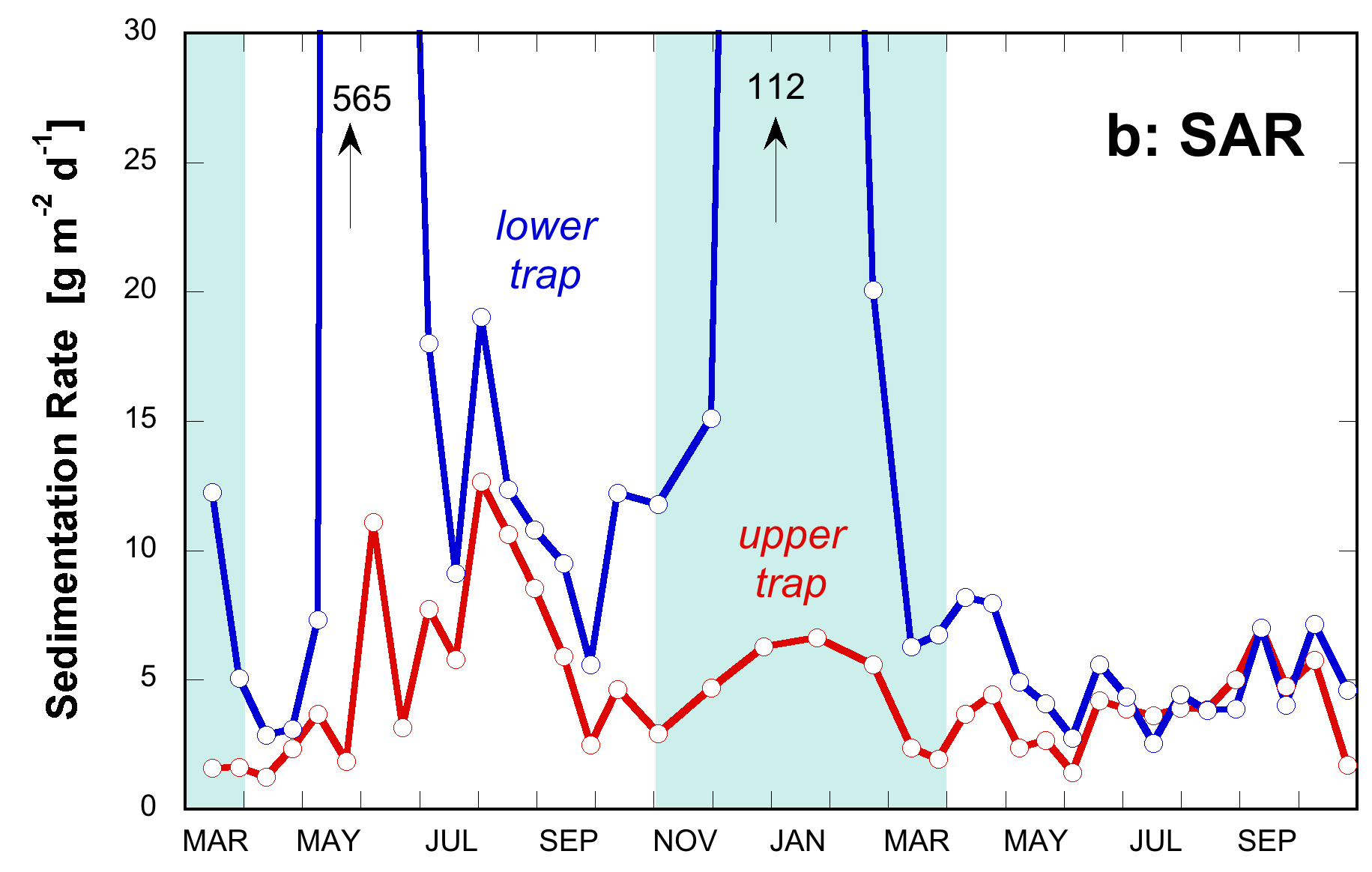

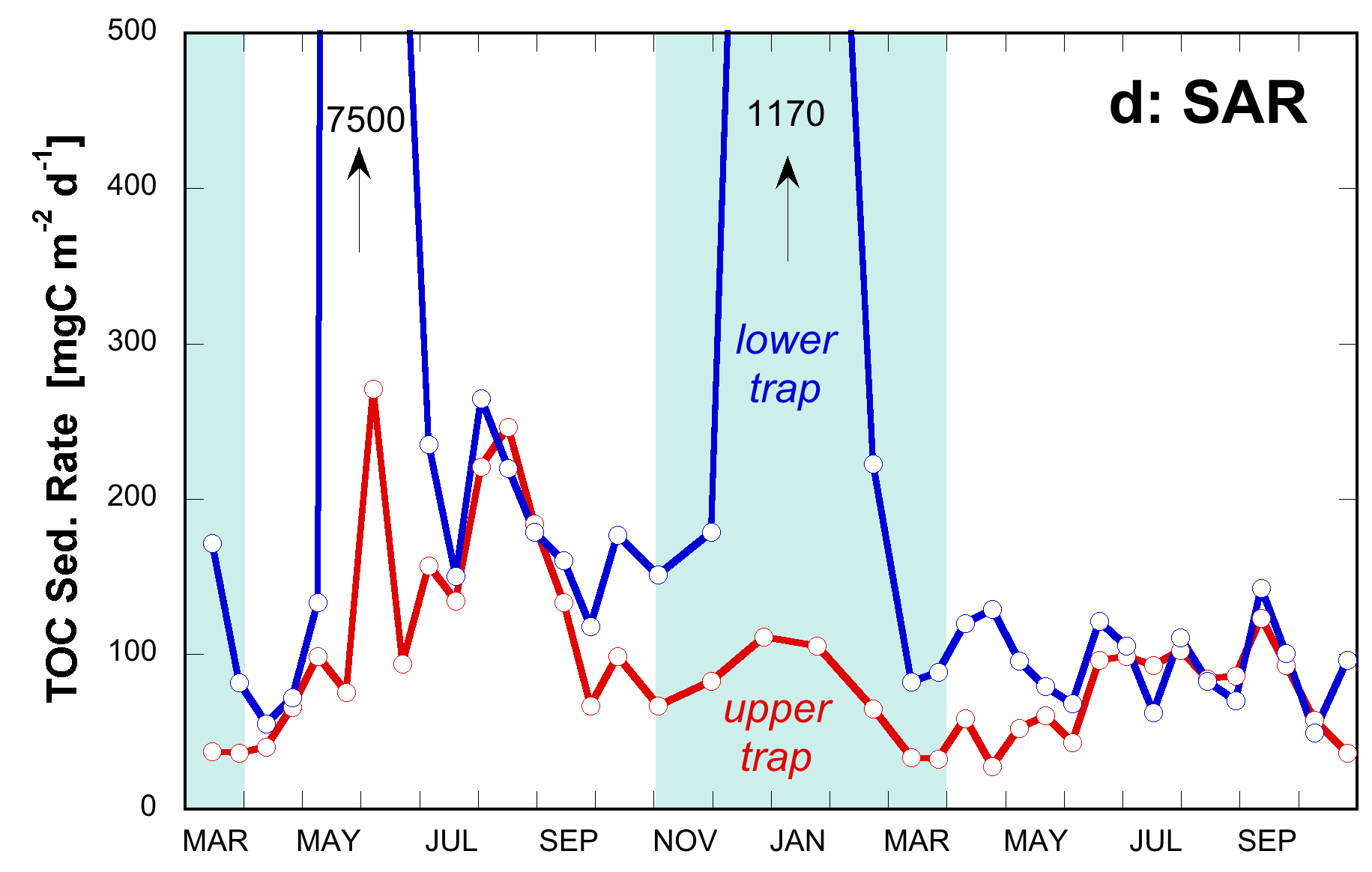


**Figure SI-1:** Carbon and nitrogen sedimentation regimes for Lakes Baldegg and Sarnen from 2017 to 2018. Sediment traps were deployed at stations A (Fig. 1 of the manuscript). The upper sediment trap (red) and the lower sediment trap (blue) were positioned at 15 m water depth, and 3 m above the sediment, respectively. Elemental C:N ratios of settling organic matter are shown in panels g and h. Dots indicate individual measurements while lines are the running average over three measurements. Time periods of winter overturn (shaded) and the bloom periods are indicated, which were also evidenced by water column nutrient profiles (Fig. SI-2).

22 mg N m^-2^ d^-1^ in September (Fig. SI-1d, f). The increase in export rates towards the end of the summer period also coincided with a slight, albeit noisy decrease in the C:N ratio reaching ~7 in September (Fig. SI-1h), which was diagnostic of active N assimilation into biomass relative to carbon.


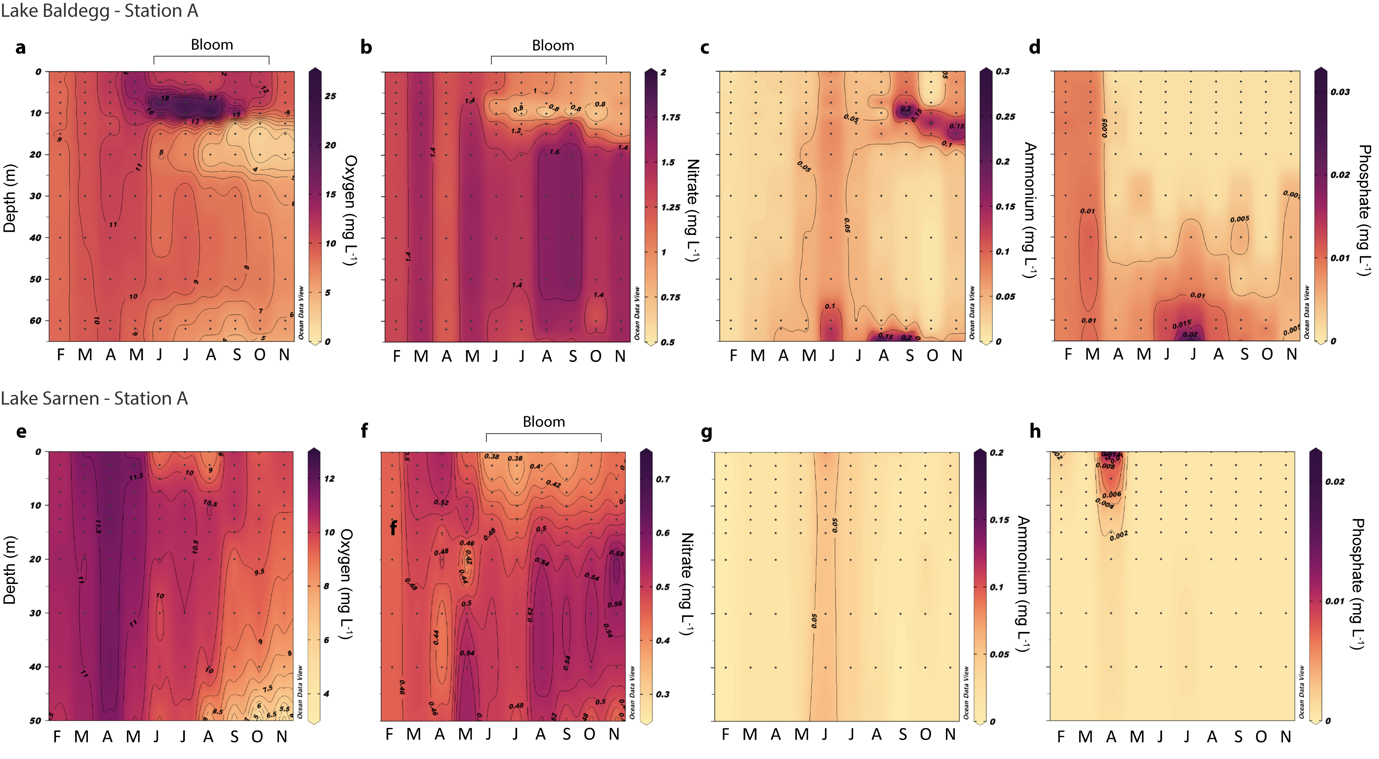


**Figure SI-2:** Contour plots of water column concentration profiles of O_2_, NO_3_^-^, NH_4_^+^, and TP of Lakes Baldegg and Sarnen in the year 2018.

**Figure SI-3:** Sediment contents of total organic carbon (a) and total nitrogen (b) from dated sediment cores of Lakes Baldegg (red) and Sarnen (blue). For Lake Baldegg, the net sedimentation data, including the TOC and TN analysis of the sediments, was previously characterized by Steinsberger et al. (2017).

**Figure SI-4:** Oxygen concentrations a few cm above the sediment-water interface (a, b), and oxygen penetration depth as measured with the micro-optode in sediment cores immediately after retrieval.


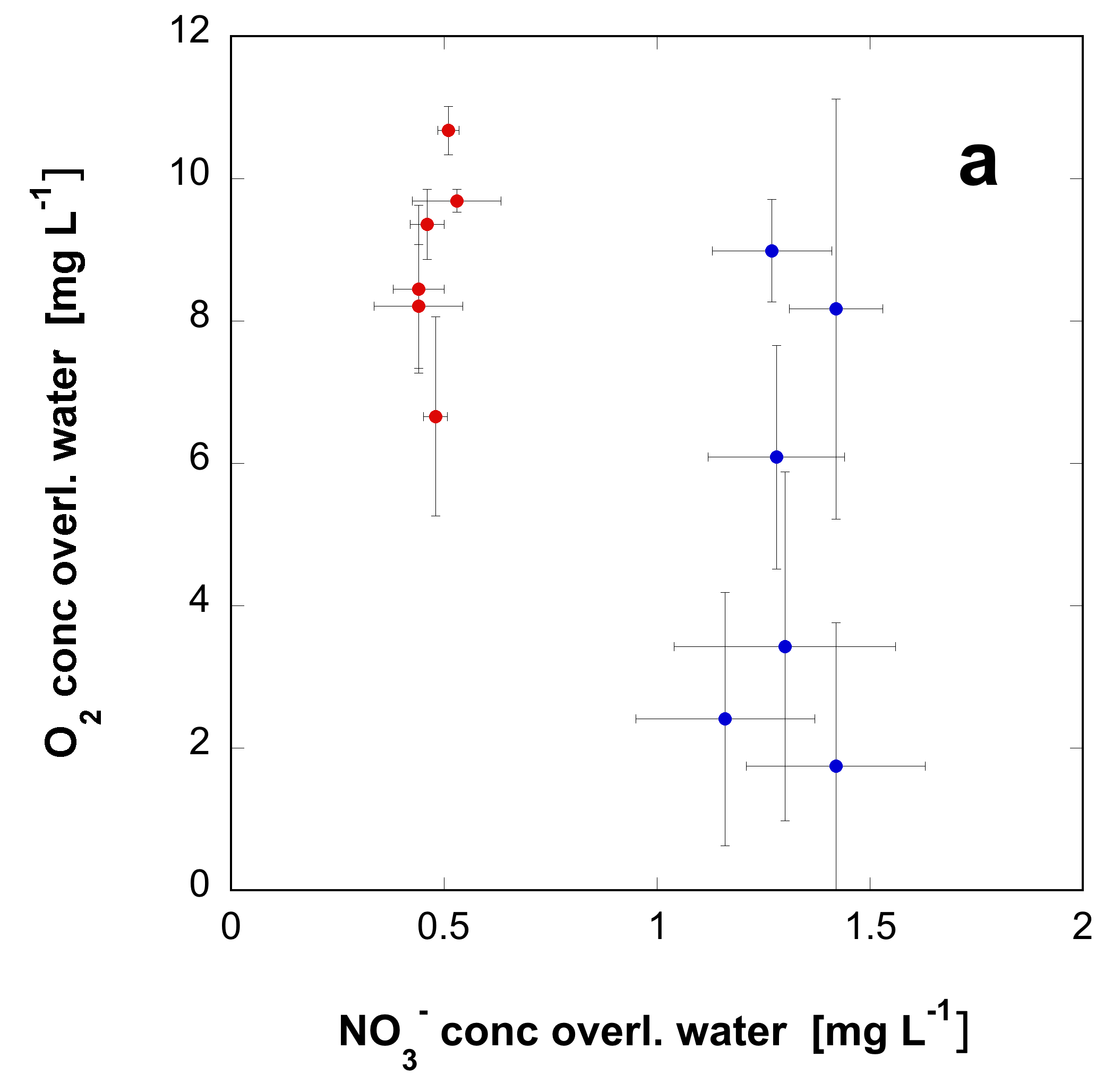

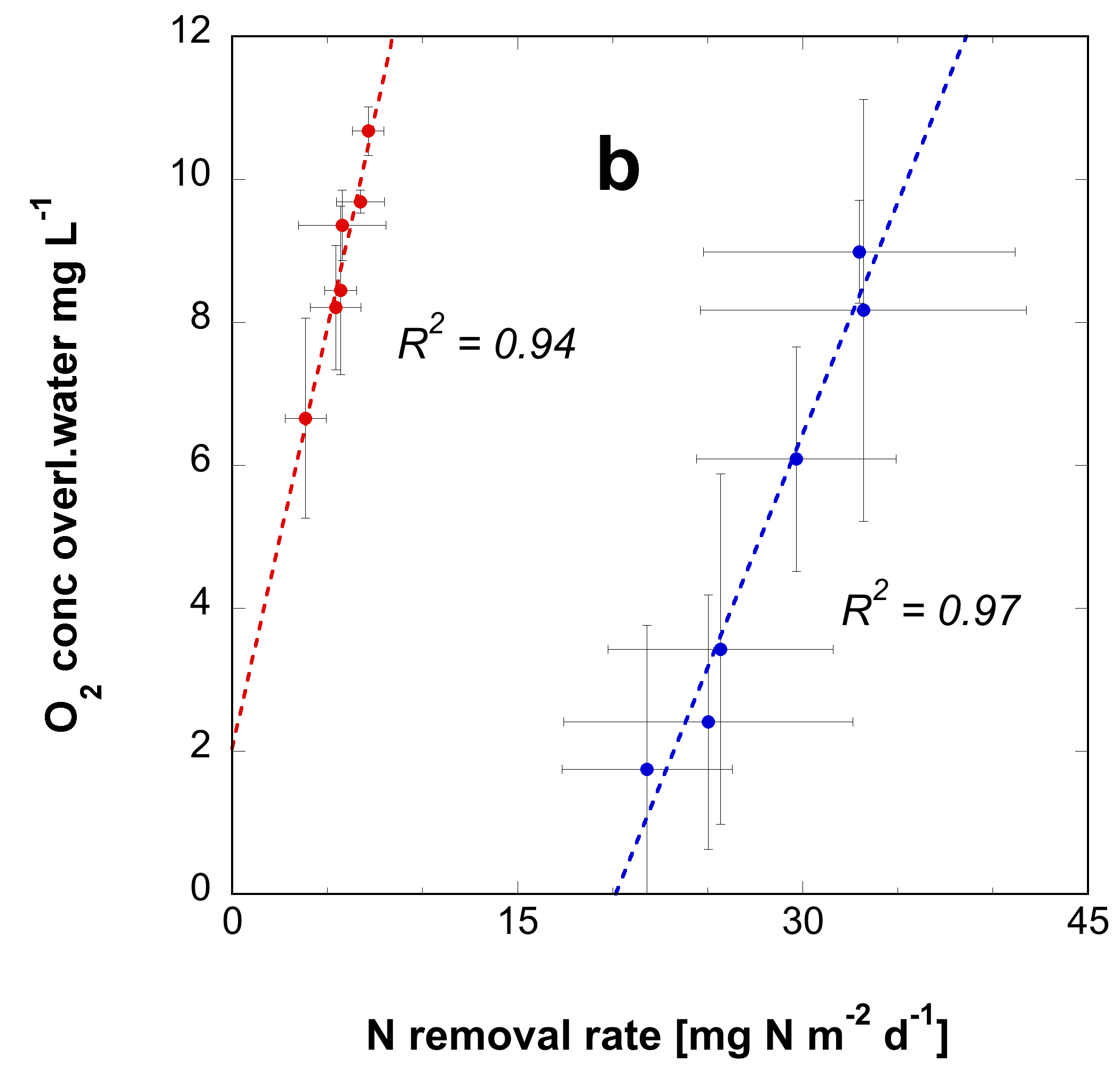


***Figure SI-5:*** *Concentrations of O_2_ and NO_3_^-^ in the sediment-overlying water averaged from all sites sampled for porewater measurements at one sampling date in Lakes Baldegg (blue) and Sarnen (red). a)* While O_2_ concentration in the sediment-overlying water varied between spring and autumn, NO_3_^-^ concentrations remained relatively constant. *b) Correlation between NRR and [O_2_] of the sediment-overlying water.*

**Table SI-1:** General overview of basic data applied in the model MODIFFUS (Hürdler et al. 2015)

| **Category** | **Characterization** | **Parameters** |
| --- | --- | --- |
| Areal statistics | land use distribution |  |
| Agricultural activity farmyards | statistic data | Number of animals, distribution, crop cultures |
| Add. Acquisition | Farmyards | Intertillage, Winter cultures |
| swissBoundaries | Borders, frontiers | Administrative boundaries |
| Chatchments | Surface waters | Hydrological catchments |
| swissTLM | Topographic landscape model | Surface waters |
| Water courses | Gauge data | Water discharge, suspended particules, manure |
| Climate | Precipitation, Evaporation | Daily precip., ann. evapor. |
| Deposition | Nitrogen deposition | Annual deposition |
| Drainage | Digitized map of cantons |  |
| Soils map |  | Soil properties |
| Erosion risk map |  | Potential erosion risk |
| Connectivity |  | Potential degree of connect. |
| Sewage treatment inventory | N & P loads |  |

## Reference:

Steinsberger T, Schmid M, Wüest A, Schwefel R, Wehrli B, Müller B (2017) Organic carbon mass accumulation rate regulates the flux of reduced substances from the sediments of deep lakes. Biogeosci 14:3275–3285. https://doi:10.5194/bg-14-3275-2017.
